# Studying the Wettability and Bonding Properties of Acetylated Hornbeam Wood Using PVAc and PUR Adhesives

**DOI:** 10.3390/ma16052046

**Published:** 2023-03-01

**Authors:** Fanni Fodor, Miklós Bak

**Affiliations:** Institute of Wood Technology and Technical Sciences, Faculty of Wood Engineering and Creative Industries, University of Sopron, Bajcsy-Zsilinszky Str. 4, 9400 Sopron, Hungary

**Keywords:** bonding, adhesive, wood modification, acetylation, hornbeam, wetting, microscopy

## Abstract

The present study aimed to determine how acetylation affected the bonding properties of European hornbeam wood. The research was supplemented with the investigation of wetting properties, wood shear strength, and microscopical studies of bonded wood, as these all have strong relationships with wood bonding. Acetylation was carried out on an industrial scale. Acetylated hornbeam showed a higher contact angle and lower surface energy than untreated hornbeam. Although the adhesion of the acetylated wood surface is lower due to its lower polarity and porosity, the bonding strength of acetylated hornbeam was similar to that of untreated hornbeam when bonded with PVAc D3 adhesive, and it was higher in the case of PVAc D4 and PUR adhesives. Microscopical studies proved these findings. After acetylation, hornbeam could be used in applications where it is exposed to moisture, as its bonding strength after soaking or boiling in water was significantly higher than that of untreated hornbeam.

## 1. Introduction

There are many fields of application for acetylated wood and wood-based panels where the bonding quality is of the utmost importance. In order to justify long-lasting bonding performance, it can be tested similarly to the shear strength of solid wood. Here, the aim is to have an adequately high bonding strength depending on the conditions of application and an increased percentage of wood failure [1]. Bonding strength is more intense in earlywood than in latewood [2].

The present study chose polyvinyl acetate (PVAc) and polyurethane (PUR) adhesives to examine the bonding properties of acetylated hornbeam wood. The wood industry uses both adhesives widely because they form flexible bonds that adapt to dimensional changes under different climatic conditions and provide high bonding strength. On the other hand, PUR can be used for structural purposes, while PVAc cannot. PVAc hardens through polymerization (loss of water), leading to a flexible linear chain. It also has a flexible aliphatic backbone. Some formulations have limited cross-linking, enabling interfacial strain due to dimensional changes in the wood that are readily distributed through the adhesive. One-component polyurethanes consist of flexible (soft) polyether or polyester segments joined together by a reaction with diisocyanates, which react with the hydroxyl (OH) groups of the adherent surfaces via CO_2_ disposal [3]. These polyurethanes retain their strength even in wet conditions, which is optimal for acetylated wood.

The hygroscopicity and hydrophilicity of wood strongly influence its bonding performance. The better the adhesive wets the wood surface, the more it penetrates the material, and the more links develop between the resin and the wood surface, ensuring a higher bonding strength [4].

Adhesive penetration is defined as the movement of fluid glue from the surface into the voids and porous structure of wood tissue, and it can be classified as “gross penetration” (mainly filling cell lumens and large voids) or “cell wall penetration” (into the tiny voids and microstructure of wood cell walls). There are several mechanisms involved in adhesion, such as mechanical interlocking, covalent bonding, and secondary interactions (e.g., hydrogen bonds), which adhesive penetration can influence. Factors related to the fluid properties of the resin, anatomical characteristics, wood permeability, and processing conditions influence the degree of adhesive penetration into wood tissue [5]. If the adhesive is manually applied to the wood material, it has deeper penetration on the bottom side where it is first applied, which allows more time for adhesive penetration [6].

Several methods can monitor adhesive penetration [7]: light microscopy of cross sections [6,8], scanning thermal microscopy [9], scanning electron microscopy (SEM) [8,10], fluorescence microscopy [11,12], porosimetry [13], neutron radiography [14], or neutron activation analysis [15], etc. Penetration behavior can be described by maximum penetration depth, but this is more precise in softwood species [10,16,17]. Here the adhesive-filled tracheids are visible as a more or less interconnected zone. Hardwood species tend to have vessels filled with adhesive isolated from the bondline. Thus, the penetration can also be described by the saturation of the accessible pore space [10,17,18], defined as the ratio of the adhesive amount and sample porosity without the adhesive. Hardwoods are more difficult to bind than softwoods because they have fibers with narrow lumens and thicker cell walls that hinder adhesive flow [19].

When the properties of wood are modified, improved durability against decaying organisms and higher dimensional stability can be achieved. On the other hand, new issues can arise, e.g., difficulties with adhesion. Using fluorescence microscopy, Bastani et al. studied the penetration of PUR and PVAc in modified wood and discovered that furfurylation decreased the effective penetration of PUR and PVAc in Scots pine [20]. On the other hand, heat treatment at 195 °C and 210 °C increased the effective penetration of PUR in beech and Scots pine. With PVAc, the effective penetration increased in heat-treated beech and Scots pine at 195 °C but was lower at 210 °C compared to untreated wood.

Due to acetylation, the wood surface becomes more hydrophobic and less polar, which leads to a higher water contact angle compared to untreated wood [21,22,23,24,25]. Bryne and Wålinder found that wetting the acetylated samples led to no significant change after the samples were conditioned for 30 days at room temperature [26]. As the acetylated wood surface has a lower water uptake, it absorbs less water-based or moisture-curing adhesives, which can lead to lower penetration and bonding strength. On the other hand, its bonding strength is less affected by dimensional changes or tension caused by moisture. Acetylated wood bonding was reported to be enhanced by a primer [27] and sanding [28].

The change in mechanical properties after acetylation is not unequivocal. The lower moisture uptake and higher density can increase the mechanical properties of acetylated wood [29,30,31]. On the other hand, permanent cell wall swelling takes place, which reduces the fiber proportion of the cross-section, and thermo-chemical degradation also takes place, which all can reduce the mechanical properties of acetylated wood [32,33]. In related literature, acetylated wood usually had a higher bonding strength and a higher rate of wood failure in dry conditions. In wet conditions, it had lower bonding strength but a higher rate of wood failure compared to untreated wood [34,35].

Initially, it was generally concluded that the acetyl groups hinder wetting and hydrogen bond formation, which cause bond failure [36,37]. Later, it was found that various factors control the performance of acetylated wood bonds [35,38].

Various studies followed up on the bonding of acetylated wood with different adhesive types, and they all concluded that PUR [28,39], resorcinol-formaldehyde, phenol resorcinol-formaldehyde, and epoxy [27,39,40,41,42,43,44,45] give satisfactory results for acetylated wood bonding. On the other hand, using emulsion polymer isocyanate, melamine-formaldehyde, and polyvinyl acetate had lower bonding strengths after acetylation [40,42,43].

Some researchers found no notable difference between adhesive penetration in untreated and acetylated wood [39], while others reported poorer adhesive penetration after acetylation [46,47].

According to Uzun et al., the bonding strength of natural hornbeam is 11.06 ± 0.14, 10.15 ± 0.17, and 9.93 ± 0.10 MPa when using polyurethane (PUR), polyvinyl acetate (PVAc-D4), and melamine formaldehyde (MF), respectively [48]. Konnerth et al. stated that higher-density species like hornbeam tend to have higher tensile strength and higher variability in strength values. Hornbeam showed considerable bonding strength loss of lap-joint samples in wet conditions and achieved low performances during delamination tests. Among tested adhesives, phenol-resorcinol-formaldehyde exhibited the highest bonding strength values [49].

After acetylation, hornbeam pH decreased from 5.11 to 4.73, and the buffering capacity increased from 1.11 mg/g to 2.15 mg/g [50], which can influence the reaction and curing time of acid-curing adhesives [39].

The present research examined bonding property changes in industrially acetylated hornbeam wood. Investigating the compatibility of adhesives and modified wood is crucial to determining how the change in wood chemistry affects bonding characteristics. Several tests were performed to draw reliable conclusions regarding this complex property. These included testing surface wettability, assessing the shear strength of solid wood samples, determining the bonding strength of samples bonded with different adhesives, and conducting microscopical studies of bonded samples.

## 2. Materials and Methods

### 2.1. Material Preparation

European hornbeam boards with the dimensions 28 × 160 × 2500 mm^3^ (T × W × L) were ordered from a Hungarian sawmill. The wood material was from the southwest part of Hungary. Half of the boards were acetylated at Accsys Technologies (Arnhem, The Netherlands) under industrial conditions [51].

### 2.2. Wettability

Untreated and acetylated hornbeam samples were prepared with radial and tangential surfaces. They were conditioned at 20 °C and 65% relative humidity until they reached a constant weight. The weight percentage gain (WPG) of the acetylated hornbeam was 15.42 ± 0.69%. The average densities of the untreated hornbeam and the acetylated hornbeam were 745 ± 6 kg/m^3^ and 757 ± 47 kg/m^3^, respectively.

The Sessile Drop Technique was used with water (polar) and diiodomethane (DIM) (nonpolar) as test liquids. The surface energies of distilled water (polar and dispersive) and diiodomethane (dispersive) are 72.8 mN/m and 50.8 mN/m, respectively. The polar component of distilled water is 46.4 mN/m (63.74%), and the dispersive component is 26.4 mN/m (36.26%).

Before measuring the contact angle, the surfaces were sanded in the same way as if they were to be bonded with adhesive (EN 205: 2016). A PGX goniometer was used for the test. The volume of the probe liquids was 5 μL, and the contact angles were determined at 0, 0.1, 1, 2, 5, 10, 20, 30, 40, and 50 s. The surface energy was calculated by the Owens–Wendt equation [52].

### 2.3. Shear Strength

The shear strength was determined according to MSZ 6786-6:1977 in the conditioning sequences (CS) defined by EN 204:2016:

- CS 1: seven days in a standard atmosphere (20 °C and 65% RH);
- CS 3: CS 1, four days in cold water at 20 °C;
- CS 4: CS 3, seven days in a standard atmosphere (20 °C and 65% RH);
- CS 5: CS 1, six hours in boiling water and 2 h in cold water at 20 °C.

The shear strength was tested with an Instron 4208 universal testing machine, and a Memmert WNB 7-45 water bath was used to heat the samples. The testing speed was 1 mm/min. There were at least seven samples of each species for each conditioning sequence.

### 2.4. Bonding Strength

Bonding strength was determined according to EN 205:2016, using beech, hornbeam, and acetylated hornbeam standard samples. The densities of beech, untreated hornbeam, and acetylated hornbeam were 658 ± 41 kg/m^3^, 725 ± 45 kg/m^3^, and 794 ± 49 kg/m^3^, respectively. The adhesives were a PVAc D3 adhesive (‘Ponal Super 3’), a PVAc D4 adhesive (‘Ponal Super 3’ + 5% ‘Ponal D4 Hardener’), and a one-component PUR adhesive (‘Ponal Pur-Leim’), all supplied by Henkel Magyarország Ltd. (Table 1). They were applied on the tangential surface, where 0.5 N/mm^2^ pressure was applied for at least 1 h at 20 °C and 65% relative humidity. The bonding strength was tested using a Tinius Olsen H10KT universal testing machine. A Memmert WNB 7-45 water bath was used to heat the lap joint samples. The durability classes were determined according to EN 204:2016. There were 20 samples of each species for each conditioning sequence (CS 1, 3, 4, and 5). The testing speed was 50 mm/min.

### 2.5. Microscopic Evaluation

Cubes with 10 × 10 × 10 mm^3^ dimensions were cut from the sample ends and dried at 103 °C in a drying kiln to achieve a constant weight. Afterward, they were placed in a desiccator. The cubes were cut with a circular saw, which resulted in rough surfaces. Hence, the surfaces were smoothed with a razor blade before examination with a Hitachi S-3400N PC-Based Variable Pressure Scanning Electron Microscope (Hitachi, Tokyo, Japan) and its software (version 1.24 volt (serial number: 340632-01)). Cross and longitudinal sections were also examined. Microscopic analysis was performed at a 60 Pa vacuum and a 10 kV accelerating voltage using a BSE detector. The working distance was 10 mm. The surfaces were not coated with a sputter coater before imaging. SEM was used due to its high level of magnification and greater depth.

The microscopic images were examined manually in AutoCAD software 2019 to measure dimensions and area. The field of view was 1100 × 600 µm [20,53]. Adhesive penetration was quantified by effective penetration (EP) and maximum penetration (MP) [6]. Effective penetration is the total area of adhesive detected in the interphase region of the bondline divided by the width of the bondline. Maximum penetration is the average penetration distance of the five most distant adhesive objects detected within the field of view.

### 2.6. Statistical Analysis

Statistical analysis was performed using the Dell Statistica software (version 13, Dell Inc., Round Rock, TX, USA). A factorial analysis of variances (ANOVA) combined with the Fisher’s LSD test was conducted, and the differences were considered significant at *p* < 0.05.

## 3. Results and Discussion

### 3.1. Wettability

Table 2 displays the test results, with data measured at 1 s. Acetylated hornbeam had a higher water contact angle and lower surface energy compared to untreated hornbeam, which corresponds to its lower water uptake and higher dimensional stability [51]. Other researchers also reported a higher water contact angle after acetylation [21,22,23,24,25], e.g., the water contact angles of unmodified and acetylated (WPG 24%) radiata pine were 45° and 98°, respectively [21].

**Figure 1:**
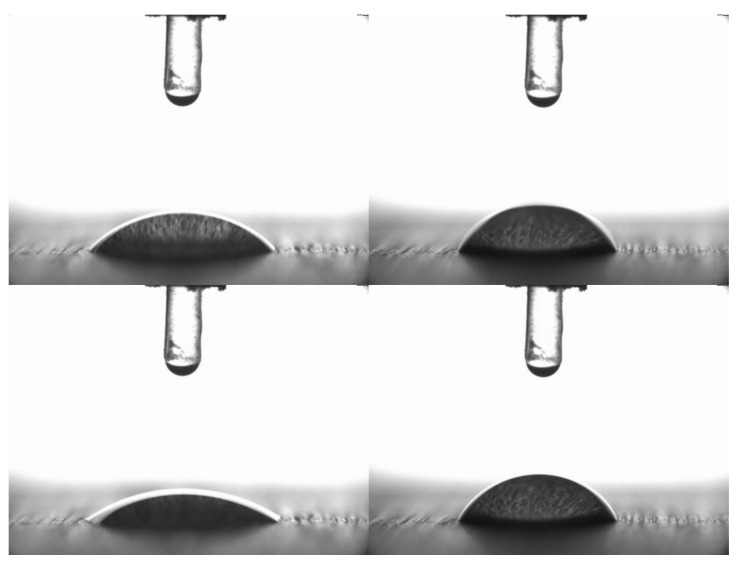
A water drop on untreated (**left**) and acetylated (**right**) hornbeam, at 0 s (**above**) and 50 s (**below**). The field of view was 7.0 mm wide and 5.2 mm high.

These numbers correspond to our results, where the contact angle of water increased from 43–44° to 61–62° (WPG 15%), which is more than a 40% increase after acetylation. Water penetration time was longer (Figure 1). The polar component of the surface energy is reduced by more than half after acetylation.

The dispersive component makes up the greater part of the surface energy, which is 75% in hornbeam and 87% in acetylated hornbeam.

The large aggregate rays occasionally present in hornbeam do not influence the wetting characteristics, as there was no significant difference between the wettability of the tangential and radial surfaces.

The water contact angle significantly changed after acetylation in both anatomical directions. The difference between the water contact angles of the radial and tangential surfaces of acetylated hornbeam was statistically significant. The results for diiodomethane were not significantly different for untreated and acetylated hornbeam.

### 3.2. Shear Strength

Table 3 contains the shear strength test results, which reveal that CS1 shear strength decreased by 9% after acetylation. This phenomenon can be explained by cell wall degradation caused by the increased temperature and pressure, and the presence of acetic acid during acetylation.

On the other hand, due to its lower moisture uptake, acetylated hornbeam had a higher shear strength compared to untreated hornbeam in moist conditions (CS3, CS4, and CS5). After soaking the samples in cold water for four days (CS3), the shear strength was higher by 114% after acetylation. A similar tendency can be found after another seven days of conditioning (CS4), where the shear strength was higher by 17% after acetylation. After soaking in boiling water for six hours and in cold water for two hours (CS5), the shear strength was greater by 81% after acetylation.

After acetylation, shear strength changed significantly in moist conditions. It also had a higher deviation than untreated hornbeam. Previous studies encountered similar findings [51]. Acetylated hornbeam had stiff fractures, while untreated hornbeam had clean breaks, indicating that, due to lower hygroscopicity and chemical alterations in the microstructure [50,54], hornbeam became more tough and flexible after acetylation.

### 3.3. Bonding Strength

Beech species were added to the tests in order to see whether the adhesive met the standard requirements or not. These limit values in the standard are defined for beech only. The bonding strengths of untreated and acetylated hornbeam can be compared to assess the effect of chemical modification on the bonding properties. Table 4 summarizes the results.

In cases of beech and hornbeam wood, there were a high number of samples where soaking resulted in sample twisting, influencing the credibility of the test.

The bonding strengths of beech, hornbeam, and acetylated hornbeam bonded with PVAc D3 adhesive met the requirements for D3 durability grading. Beech and hornbeam have a similar, diffuse-porous microstructure, which enables easy bondability. On the other hand, hornbeam had the worst bonding properties, having the greatest bond strength reduction after soaking (CS3, −83%) and after conditioning (CS4, −19%). After acetylation, the bonding strength increased by 65% in wet conditions (CS3), but it had a weaker bonding strength after conditioning (CS1 and CS4). Similar results were found in the literature [40,42,43]. Untreated and acetylated hornbeam both displayed a high incidence of glue line failure, revealing that the wood was stronger than the bondline.

Beech and hornbeam samples bonded with the PVAc D4 2-C adhesive failed to meet D4 grading according to the standard in wet conditions (CS3 and CS5). In this case, most of the specimens broke in the bondline. These results could be explained, e.g., by the wrong viscosity of the adhesive mix, leading to reduced bonding quality. Hornbeam had the lowest reduction in bonding strength after soaking in cold water (CS3, −62%) but the greatest after soaking in boiling water (CS5, −78%). Because hornbeam had lower permeability than beech, their bonding strength results could differ [55]. Acetylated hornbeam met the D4 standard requirements. Acetylation improved the bonding strength of hornbeam after conditioning (CS1, +39%) and soaking in boiling water (CS5, +153%).

All three species that bonded with the “PUR” adhesive met the standard requirements for D4 grading. Hornbeam had similar (CS1 and CS3) or worse (CS5) bonding strength results compared to beech. It had the largest bonding strength reduction after soaking in cold water (CS3, −66%) and boiling water (CS5, −73%), while acetylated hornbeam had the lowest reduction (CS3, −37% and CS5, −38%, respectively). Acetylation greatly increased the bonding strength of hornbeam when soaked in cold water (CS3, +63%) and boiling water (CS5, +103%).

The PUR adhesive exhibited a higher bonding strength than PVAc in dry conditions, and it also retained its strength better when exposed to water. This corresponds to other studies [28,39,48]. Thus, PUR is a suitable choice for exterior products made of acetylated hornbeam.

In an Accoya^®^ study, PUR-bonded Accoya^®^ had a low delamination percentage and met the standard requirements. The bonding strength in wet conditions decreased by 13–27% and 65% for acetylated and untreated radiata pine, respectively. The wood failure of acetylated radiata pine failed to meet the standard requirements in dry conditions. Acetylated radiata pine laminates bonded with PVAc showed poor performance in the delamination tests compared to those bonded with PUR [39].

A high percentage of wood failure indicates greater adhesive penetration and interfacial bonding between the wood and the adhesive [56], which was true for the conditioned samples.

After acetylation, hornbeam dimensional stability (anti-swelling efficiency) increased to 81–88% [51], which has a direct influence on reducing stresses on bondlines in delamination tests associated with water immersion and drying cycles. Furthermore, compared to untreated wood, the mechanical properties decreased less in wet conditions, indicating a higher bonding strength in wet conditions as well [57,58].

The shear strength of solid wood was significantly higher than the bonding strength of the same species (at the same conditioning sequences), but with a similar trend of change.

Statistically significant differences can be found in the results where acetylation increased the bonding strength of hornbeam wood (Table 4). When comparing the results of different adhesives, PUR exhibited a significantly higher bonding strength than PVAc.

### 3.4. Microscopic Evaluation

Penetration is mainly influenced by adhesive characteristics and wood microstructure. Hornbeam is a diffuse-porous species in which the adhesive flows through longitudinal vessels and proceeds along paths of lowest resistance toward the wood tissue [59]. Typical bondline imperfections are bondline starvation for PUR due to the high adhesive mobility and void formation inside the bulk adhesive in PVAc due to excessive shrinkage [17]. Table 5 lists the results of effective penetration and maximum penetration for untreated and acetylated hornbeam.

**Figure 2:**
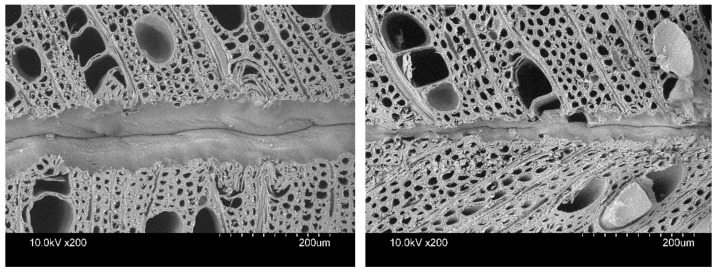
Micrographs of D3 PVAc-bonded untreated (**left**) and acetylated (**right**) hornbeam wood. A thinner bondline, greater penetration, and more small voids can be observed in acetylated hornbeam than in untreated hornbeam.

PVAc is a water-based, water-dispersed adhesive that may reach an adhesion optimum when the water has penetrated the wood substrate [60]. PVAc has good flow into cell lumens, but its high molecular weight prevents it from penetrating cell walls; thus, only gross penetration can be observed [61]. According to a related study, chemical modification (furfurylation) can decrease PVAc bondline thickness and penetration depth [20]. On the micrographs of PVAc D3-bonded wood, the lumens and large vessels were rich in adhesive. Acetylated hornbeam had a lower effective penetration and a thinner bondline (Figure 2). These correspond to the fact that its bonding strength was similar to untreated hornbeam and that it had a low percentage of wood failure, indicating poorer bonding.

**Figure 3:**
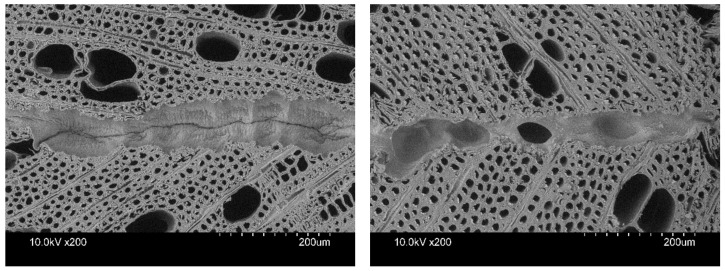
Micrographs of D4 2-C PVAc-bonded untreated (**left**) and acetylated (**right**) hornbeam wood. Thin bondlines and low penetration can be observed on both untreated and acetylated hornbeam.

PVAc D4 had a lower effective penetration and a thinner bondline after acetylation, but the adhesive penetrated deeper into the wood than the untreated hornbeam (Figure 3), which may explain its enhanced bonding strength and high incidence of wood failure. The results of PVAc D3 are higher in both effective penetration and maximum penetration compared to PVAc D4.

**Figure 4:**
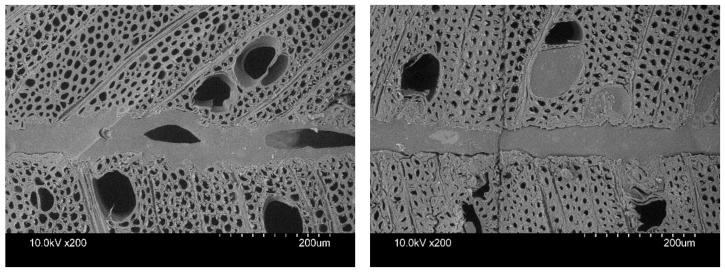
Micrographs of PUR-bonded untreated (**left**) and acetylated (**right**) hornbeam wood. Hornbeam shows signs of overpenetration with starved bondlines and voids. This was not observed in acetylated hornbeam.

PUR exhibited the highest bonding strength and greatest effective penetration in acetylated hornbeam, which is attributable to its lower molecular weight compared to PVAc. It can also be explained by the fact that there are fewer available functional hydroxyl groups in acetylated hornbeam, which enabled fewer chemical reactions with the isocyanate ingredient of PUR. There were signs of overpenetration in untreated hornbeam, with starved bondlines and voids in bondlines (Figure 4) [10]. Acetylated hornbeam had greater effective penetration, lower penetration depth, and no signs of starved bondline, which corresponded to the literature [4,20]. In a similar study, the effective penetration was 76.06 µm in beech and 96.87–99.09 µm in heat-treated beech [20].

PVAc D3 achieved the greatest effective penetration in hornbeam, while PUR achieved the same for acetylated hornbeam. After acetylation, the effective penetration was higher for PUR than PVAc, while the maximum penetration was lower, matching the conclusions of a similar study on modified beech [20].

After acetylation, effective penetration changed by −37% in PVAc D3, −32% in PVAc D4, and +52% in PUR adhesives. Maximum penetration changed by +56%, +55%, and -39% in PVAc D3, PVAc D4, and PUR adhesives, respectively.

The change in effective penetration and maximum penetration after acetylation and the difference between the effective penetrations of different adhesives were all statistically significant.

## 4. Conclusions

The present study aimed to determine how acetylation affected the bonding properties of European hornbeam wood. The research was supplemented with the investigation of wetting properties, wood shear strength, and microscopical studies of bonded wood, as these all have strong relationships with wood bonding.

Acetylated hornbeam showed a higher water contact angle (+40%) and lower surface energy (−14%, with a −55% reduction of the polar component) than untreated hornbeam. This finding corresponds to the related literature and connects to the lower equilibrium moisture content, water uptake, and better dimensional stability of acetylated hornbeam.

Bonding strength limit values given by EN 204:2016 were achieved by PVAc D3 and PUR (D4) adhesives, while PVAc D4 2-c did not meet the requirements for D4 durability grading. Despite its similar diffuse-porous structure, hornbeam had similar (PUR), or lower (PVAc) bonding strengths compared to beech. Acetylation improved the properties of hornbeam wood to have similar (PVAc D3) or higher (PVAc D4; PUR) bonding strengths compared to untreated hornbeam.

Microscopical studies proved that PUR-bonded samples obtained better results than PVAc-bonded samples. In the micrographs of lap joint samples bonded with PVAc, thin bondlines and lower effective penetration but greater maximum penetration were observed after acetylation. Using PUR, the lower bonding strength of untreated hornbeam was attributed to bondline starvation and voids in the bondline.

Although the adhesion of the acetylated wood surface is lower due to its lower polarity and porosity, the bonding strength of hornbeam was not significantly reduced by acetylation. In most cases, the acetylated samples broke in the bondline, resulting in a lower proportion of wood failure.

After acetylation, the wood becomes more dimensionally stable when exposed to moisture, which enables better mechanical properties in wet conditions. Corresponding to the literature, acetylated hornbeam had significantly higher shear strength and bonding strength when soaked in water or boiling water compared to untreated hornbeam.

The shear strength of solid wood was significantly higher than the bonding strength of the same species (at the same conditioning sequences), but with a similar trend of change. Acetylated hornbeam has a somewhat weaker shear strength (−9%) and similar bonding strength (between −14% and +39%) in a dry state compared to hornbeam, but it can even double the shear of hornbeam (+17–114%) and bonding strength (+63–153%) in a soaked state.

According to these results, acetylated hornbeam can be bonded well with PVAc and PUR adhesives and can be used both in wet and humid applications. On the other hand, it requires greater caution than beech, and it is advisable to use a longer pressing time for better bonding performance.

## Figures and Tables

**Table 1:** Technical information on tested adhesives (B: beech, H: hornbeam, and AH: acetylated hornbeam).

Properties	Brookfield Viscosity (20 °C) (MPa.s)	Density (kg/m^3^)	pH	Consumption (g/m^2^)
PVAc D3 adhesive (water-based polyvinyl acetate dispersion)	9000–15,000	1060–1100	2.8–3.6	(B) 175–190 (H) 175–190 (AH) 130–150
PVAc D4 adhesive (PVAc D3 adhesive +5% modified aliphatic polyisocyanate)	2800–4000	1140–1180	n.a.	(B) 145 (H) 150–160 (AH) 150
One-component PUR adhesive (PUR prepolymer with free 4,4′-methylenediphenyl diisocyanate)	9000–14,500	1070–1140	n.a.	(B) 120 (H) 120–145 (AH) 160

**Table 2:** Contact angle and surface energy of untreated and acetylated hornbeam. Average values are presented with their standard deviations in brackets, measured at 1 s. A significant change after acetylation is marked by an asterisk if *p* < 0.05.

Wood	Contact Angle				Surface Energy					
	Water		Diiodo- Methane		All		Polar Component		Dispersive Component	
	Tan	Rad	Tan	Rad	Tan	Rad	Tan	Rad	Tan	Rad
	°				mJ/m^2^					
Hornbeam	44.02 (2.58)	43.14 (1.80)	18.26 (1.73)	18.50 (2.75)	63.83 (1.30)	64.23 (1.10)	15.57 (1.37)	16.06 (0.90)	48.26 (0.47)	48.16 (0.74)
Acetylated hornbeam	62.25 * (4.23)	61.12 * (1.76)	20.26 (3.24)	19.70 (5.28)	54.63 * (1.67)	55.32 * (1.45)	6.99 * (1.82)	7.61 * (0.99)	47.64 (0.93)	47.72 (1.54)

**Table 3:** Shear strength results of untreated hornbeam and acetylated hornbeam at different moisture states and conditioning sequences (CS) according to EN 204:2016. Average values are presented with their standard deviation in brackets. A significant change after acetylation is marked by an asterisk if *p* < 0.05.

Conditioning Sequences (CS)	Moisture Content (%)		Shear Strength (MPa)	
	Hornbeam	Acetylated Hornbeam	Hornbeam	Acetylated Hornbeam
1	12.29 (0.14)	4.68 * (0.17)	17.36 (1.26)	15.72 * (3.91)
3	52.97 (1.99)	28.39 * (1.28)	7.92 (0.50)	16.94 * (1.21)
4	16.30 (0.18)	7.45 * (0.62)	15.31 (0.87)	17.96 * (2.48)
5	79.67 (1.14)	48.32 * (2.91)	8.71 (0.23)	15.73 * (1.61)

**Table 4:** Summary of bonding strength test results for beech (B), hornbeam (H), and acetylated hornbeam (AH) at different conditioning sequences (CS) (EN 204:2016). A listing of the average bonding strength of ten lap joint samples with their standard deviation in brackets. Wood failure is given as the proportion of samples that presented an apparent cohesive wood failure during the test; conformity is given as the proportion of samples that met the standard limit (compared to the maximum number of samples, 20 pieces). A significant change after acetylation is marked by an asterisk if *p* < 0.05.

Conditioning Sequences (CS)	1			3			4			5		
EN 204:2016	≥10 MPa			≥2 MPa (D3) ≥4 MPa (D4)			≥8 MPa			≥4 MPa		
“PVAc D3”	B	H	AH	B	H	AH	B	H	AH	B	H	AH
Bonding strength (MPa)	12.4 (1.1)	12.1 (1.6)	10.4 (1.6)	2.2 (0.4)	2.0 (0.4)	3.3 * (0.3)	11.3 (1.3)	9.8 (1.5)	9.0 (1.8)	-	-	-
Wood failure (%)	70	35	15	0	0	0	95	60	0	-	-	-
Conformity (%)	70	60	35	35	20	65	90	45	40	-	-	-
“PVAc D4 2-C”	B	H	AH	B	H	AH	B	H	AH	B	H	AH
Bonding strength (MPa)	11.1 (1.1)	7.6 (3.6)	10.6 * (1.4)	3.2 (0.5)	2.9 (0.4)	2.9 (0.2)	-	-	-	3.5 (0.5)	1.7 (0.8)	4.3 * (0.3)
Wood failure (%)	50	10	25	0	0	0	-	-	-	0	0	0
Conformity (%)	50	10	30	0	0	0	-	-	-	15	0	50
“PUR”	B	H	AH	B	H	AH	B	H	AH	B	H	AH
Bonding strength (MPa)	11.6 (0.9)	12.9 (1.2)	11.4 (1.7)	4.6 (0.6)	4.4 (0.3)	7.2 * (0.4)	-	-	-	4.1 (0.3)	3.5 (0.7)	7.1 * (0.5)
Wood failure (%)	65	35	70	0	0	0	-	-	-	0	0	25
Conformity (%)	60	90	40	55	45	80	-	-	-	30	10	70

**Table 5:** Effective penetration and maximum penetration results of untreated and acetylated hornbeam bonded with PVAc and PUR adhesives. Average values are presented with the standard deviation in brackets. A significant change after acetylation is marked by an asterisk if *p* < 0.05.

Adhesive	Material	No. of Sections	Effective Penetration (µm)	Maximum Penetration (µm)
PVAc D3	Untreated hornbeam	7	106 (8)	114 (40)
	Acetylated hornbeam	8	67 * (14)	178 * (19)
PVAc D4	Untreated hornbeam	5	71 (4)	87 (33)
	Acetylated hornbeam	7	48 * (7)	135 * (42)
PUR	Untreated hornbeam	7	58 (4)	136 (23)
	Acetylated hornbeam	7	88 * (18)	83 * (17)

## Data Availability

Not applicable.
